# Supplementary material for: The administration of high-mobility group box 1 fragment prevents deterioration of cardiac performance by enhancement of bone marrow mesenchymal stem cell homing in the delta-sarcoglycan-deficient hamster
Source: PLoS One. 2018 Dec 5;13(12):e0202838. doi: 10.1371/journal.pone.0202838 (PMC6281303; doi:10.1371/journal.pone.0202838)
Supplement: S1 Table — (PDF) [file pone.0202838.s001.pdf]

S1 Table. Forward and reverse primers and probe

	F-primer	R-primer	Probe
GAPDH	CTG CAC CAC CAC CTG CTT AGC	GCC ATG CCA GTG AGC TTC C	CTG CAC CAC CAC CTG CTT AGC
HGF	AGG TCC CAT GGA TCA CAC AGA	GCC CTT GTC GGG ATA TCT TTC T	ACC AGC AGA CAC CAC ACC GGC A

GAPDH, glyceraldehyde-3-phosphate dehydrogenase; HGF, hepatic growth factor
